# Abnormal Alpha Rhythm During Self-Referential Processing in Schizophrenia Patients

**DOI:** 10.3389/fpsyt.2019.00691

**Published:** 2019-10-01

**Authors:** Shikui Jia, Miaomiao Liu, Peiwen Huang, Yanli Zhao, Shuping Tan, Ritsu Go, Tianyi Yan, Jinglong Wu

**Affiliations:** ^1^Intelligent Robotics Institute, School of Mechatronical Engineering, Beijing Institute of Technology, Beijing, China; ^2^Graduate School of Natural Science and Technology, Okayama University, Okayama, Japan; ^3^Center for Psychiatric Research, Beijing Huilongguan Hospital, Beijing, China; ^4^School of Life Science, Beijing Institute of Technology, Beijing, China

**Keywords:** schizophrenia patients, self-referential processing, alpha rhythm, functional connectivity, brain network

## Abstract

Schizophrenia patients exhibited a psychological abnormal appearance when they recognized objects related to themselves. This cognitive process is associated with self-referential processing. In this study, the self-referential memory (SRM) task was performed by 18 schizophrenia patients and 18 healthy controls. In the encoding stage of the SRM task, the behavioral experiment data and electroencephalogram (EEG) data were recorded in three experimental conditions (self-referential condition, other-referential condition, and physical condition). For data analysis, the electrophysiological performance of the time-frequency distribution, phase lag index (PLI) strengths, phase synchronization connectivity, and brain-network properties were assessed in schizophrenia patients compared to healthy controls. We found that schizophrenia patients exhibited abnormal alpha oscillation characteristics at the time of 100–300 ms poststimulus during the self-referential condition, which consisted of diminished time-frequency distributions over the prefrontal, parietal, and occipital regions; lower functional connectivity strengths of the PLI in the parietal and occipital areas; higher global efficiency and the lower characteristic path length; and nodal efficiency of local areas (increased nodal efficiency in temporal regions and decreased nodal efficiency in occipital region) for dynamic network topology properties. Furthermore, the evoked power of the alpha band during the self-referential condition was significantly correlated with the SRM bias score in the patients (r = 0.595, p = 0.009). These results provided electrophysiological evidence and supported the hypothesis that an abnormal alpha rhythm might be the principal factor of dysfunctional self-referential processing in schizophrenia patients.

## Introduction

An individual generates a conscious process of self-referential processing when making a decision related to one’s own self (1). In self-referential processing, the normal participant’s performance, which is self-referential memory (SRM) in the self-referential condition, is better than the other-referential condition (2, 3). However, schizophrenic patients have demonstrated distinct defects in self-referential processing (4). Harvey et al. discovered that schizophrenia patients presented a lower performance from the SRM bias (5). Abnormal self-reflection (self-referential processing) and the related brain network are associated with the expression of psychosis (6). Previous studies have demonstrated that self-referential processing occurs in connection with cortical midline structures, particularly the medial prefrontal cortex (MPFC), by functional magnetic resonance imaging (fMRI) techniques (7). Compared to healthy controls, schizophrenia patients present less activation in the posterior cingulate cortex in self and other reflection conditions using fMRI with insight tasks (8). Previous studies have demonstrated that self-referential processing plays a vital role for schizophrenia patients.

Self-referential processing in schizophrenia has been principally researched by fMRI. However, limited studies using electroencephalogram (EEG) have investigated the cognitive process of self-referential processing in schizophrenia. With a focus on normal individuals using EEG and event-related potential (ERP), the performance of self-referential conditions compared with other-referential conditions has been explored in several studies. In the occipital and temporal regions, the results with trails of pronouns related to “my” and “his” are different from 250 to 400 ms, respectively (9). With respect to studies on schizophrenia patients by ERP, a previous study identified lower P2 amplitudes occurring in paranoid schizophrenia compared to healthy controls during the self-referential condition (10), as well as different N2 amplitudes between the self-reflection condition and other-reflection condition (11). The electrophysiological activity of schizophrenia patients might contribute to the abnormality of self-reflective processing 200 ms poststimulus. In conclusion, previous studies have mainly explored self-referential processing focused on normal individuals by EEG, whereas a minority of previous studies have thoroughly investigated schizophrenia during self-referential processing.

In addition to fMRI, EEG, positron emission computed tomography (PET), and other different data acquisition techniques, different data analysis methods may also explore different aspects of neurological mechanisms. Phase synchronization is an effective method to construct the functional connectivity of EEG detection. EEG is ideal for building large functional connectivity networks and for the analysis of various frequencies, especially since it has good time resolution. Several studies have provided evidence of neural activity dysfunction in schizophrenia with phase synchronization connectivity and brain-network analysis (12, 13). However, a strong false connection is generated because of the positional deviation between the effect of the recording signal (14). As one synchronization analysis method, the phase lag index (PLI) (15, 16) was calculated between two events at the same time point estimated in statistics, which quantized the phase variability in various frequency bands. The PLI could attenuate the zero-phase difference of synchronization and reduce the impact of spurious synchronization. Furthermore, schizophrenia patients have obvious reduced functional connectivity strength measured by PLI in the alpha band compared to healthy controls (17). In this study, the PLI is calculated to construct connectivity networks according to graph theory between healthy controls and schizophrenia patients during self-referential processing.

In this study, we investigate electrophysiological activities during self-referential processing with SRM tasks in schizophrenia patients and healthy controls by EEG. The SRM task is prominent to evaluate self-referential processing in electrophysiological and behavioral experiments. In the analytical method, different frequency bands were extracted in three conditions, and the primary abnormalities in each frequency band were explored. The PLI was calculated for each pair of electrodes from the EEG data of one specific frequency band. Furthermore, the PLI, which is calculated in one specific frequency band, constructs the functional connectivity and brain network. Using functional connectivity and brain-network peculiarities, the electrophysiological activity was analyzed in schizophrenia patients compared to healthy controls. It was hypothesized that the abnormal specific oscillation is exhibited as a prominent performance during self-referential processing in schizophrenia patients, and there are significant differences compared with healthy controls.

## Materials and Methods

### Participants

Eighteen patients with schizophrenia from Beijing Huilongguan Hospital and 18 normal controls were recruited for the SRM task and EEG recording. The healthy controls were recruited through advertising around Beijing Huilongguan Hospital and advertisements on the internet. All patients were diagnosed according to the *Diagnostic and Statistical Manual of Mental Disorders, 4th Edition (DSM-IV)* (18) and Positive and Negative Syndrome Scale (PANSS) (19) and were confirmed by a senior psychiatrist. The control participants were required to make evaluations with the Structured Clinical Interview for *DSM-IV-TR* Axis 1 Disorders, Research Version, Non-Patient Edition (SCID-I/NP) (20) and Structured Clinical Interview for *DSM-IV* Axis II Personality Disorders (SCID-II) (21). Additional criteria in this experiment for schizophrenia patients: (1) no brain damage or history of sequela, (2) no depressive or manic episode during experiment, (3) IQ > 70 (22) based on medical records, (4) no history of drug dependence in the past 6 months, (5) no electroconvulsive treatment in the past 6 months, and (6) receiving drug treatment at least 1 month. Additional criteria in this experiment for healthy controls: (1) no history of drug dependence in the past 6 months, (2) no depression trend, (3) no history of psychiatric or neurological illness, (4) no brain damage or sequela, and (5) no family medical history of schizophrenia. There was no significant difference between the schizophrenia patients and healthy controls in age, educational status, sex, or handedness (**Table 1**). All participants were paid for their participation and had normal or corrected-to-normal vision. All participants performed the experiment at the scheduled time in the laboratory of Beijing Huilongguan Hospital and successfully completed the experiment. Prior to the experiment, informed consent was obtained, and the experiment was approved by the local ethics committee (Beijing Huilongguan Hospital) and was in compliance with the ethical guidelines of the American Psychological Association (APA).

**Table 1:** Statistics and clinical data for patient and control groups. Descriptive data as mean (range) or mean ± standard deviation.

	Patients	Controls	Statistics
Age (years)	35 ± 8.9	30.7 ± 6.9	F_1,34_ = 2.446, p = 0.127
Education (years)	13.4 ± 2.3	14.6 ± 2.1	F_1,34_ = 2.16, p = 0.151
Sex (male/female)	11/7	9/9	χ^2 ^= 0.45, p = 0.502
Handedness (right/left)	18/0	18/0	
Duration of illness (months)	121 (1–384)		
Age at disease onset (years)	23.3 ± 7		
PANSS score	57 ± 9.74		
Subtype, paranoid/undifferentiated	7/11		
Chlorpromazine equivalents (mg/day)	498.6 ± 283.5		

PANSS, Positive and Negative Syndrome Scale.

### Experimental Stimuli and Procedure

The experiment was performed to investigate self-referential processing through the SRM paradigm (11, 23). A total of 310 adjectives (155 positive and 155 negative adjectives), which were selected from Yang & Wang’s Personality Trait Adjective List (24), were presented in Chinese during the SRM task. The SRM task includes two stages: the encoding stage and the recognition stage. In the encoding stage, there are three conditions, which consist of the self-referential condition, other-referential condition, and physical condition. A total of 210 adjectives (105 positive and 105 negative adjectives), which were selected from Yang & Wang’s Personality Trait Adjective List, were presented in the encoding stage. In each condition, 70 adjectives (35 positive and 35 negative adjectives) were selected. In the self-referential condition, the participants were required to judge whether a series of adjectives describe themselves. In the other-referential condition, the participants were required to judge whether a series of adjectives describe other individuals. In the physical condition, the participants were required to judge whether the typesetting is in bold. After several minutes, in the recognition stage, pseudorandom adjective trials were presented, and the participants were required to judge whether the words had appeared in the encoding stage. A total of 100 adjectives (50 positive and 50 negative adjectives), which were selected from Yang & Wang’s Personality Trait Adjective List, were presented.

**Figure 1:**
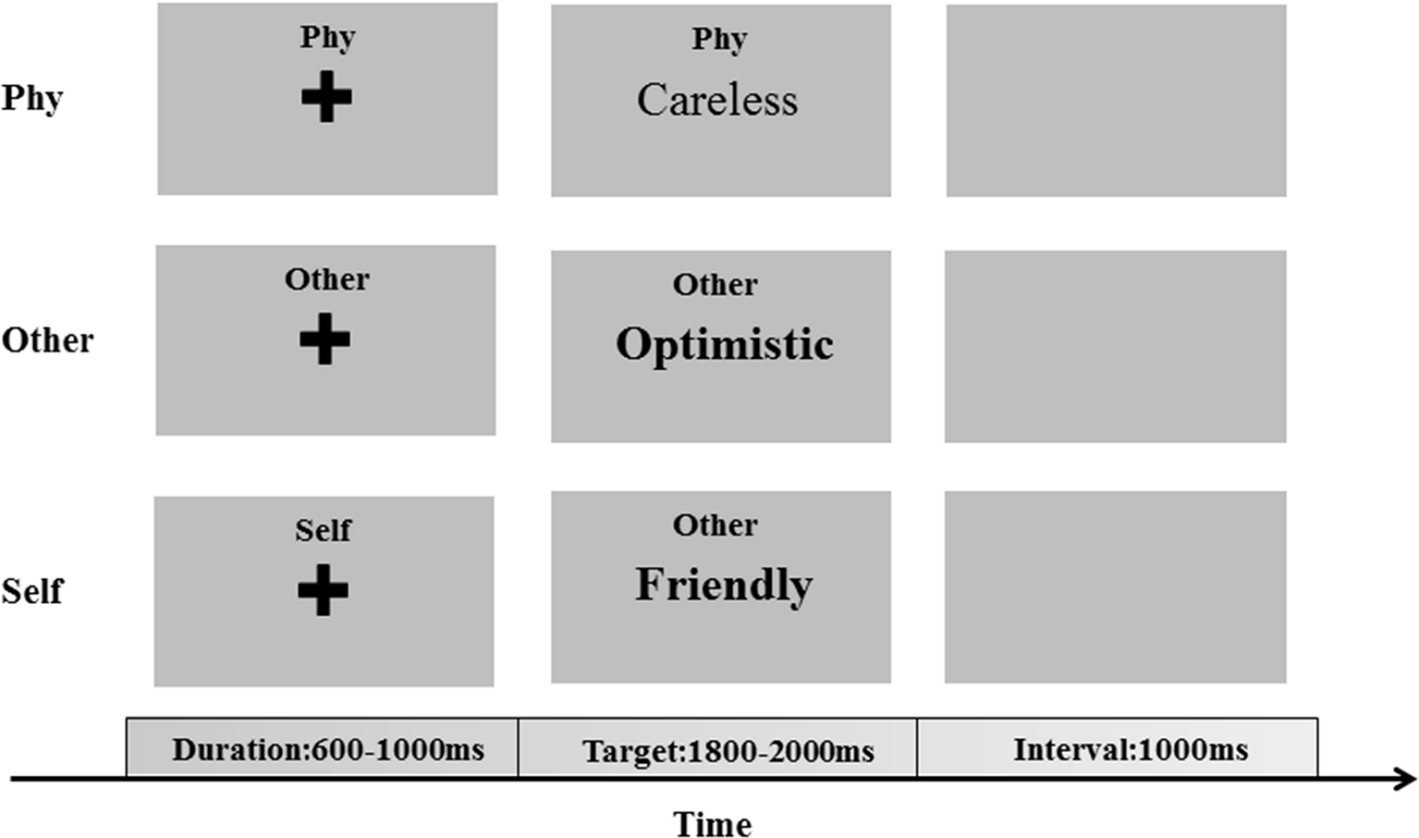
The encoding phase of the self-referential memory (SRM) task. The cues and trait adjectives were displayed in Chinese.

All stimuli were presented in the center of the screen and in black color on a gray background with the same contrast and brightness. In each trial of one condition, a fixation cross was initially presented for a duration of 600–1,000 ms, following which the target stimuli were presented with a maximum duration of 2,000 ms (**Figure 1**). The participants were required to respond to the associated question by pressing the key with their left and right index fingers. With an inter-stimulus interval of 1,000 ms, the next trial was started after the participants responded to the target stimuli.

### Behavioral Measures and Analysis

In this experiment, the stimulus display and behavioral data were recorded using E-Prime software (version 2.0, pstnet.com/products/e-prime/). The recognition score was calculated as the proportion of hits minus the proportion of false alarms in each condition (3, 25). The SRM bias score was calculated by the recognition score of the difference between the self-referential condition and the other-referential condition (11).

### Electroencephalogram Recording and Preprocessing

EEG signals were recorded using a standard 10–20 system with 64 channels (Brain Products, Germany). The left mastoid was selected as the reference electrode during the recording of EEG data, re-referenced with an averaged mastoid offline. The electrode impedance was maintained below 5 k. In addition to the reference electrode and electro-oculogram electrodes, 59-channel EEG data were analyzed.

To investigate the electrophysiological activity of self-referential processing between schizophrenia patients and healthy controls, the EEG data were recorded in the encoding stage of the SRM task. The recorded EEG data in this study were preprocessed with a freely available EEGLAB (http://sccn.ucsd.edu/eeglab/) toolbox in MATLAB R2014a (26). The band-pass filtering was employed from 0.1 Hz to 30 Hz. Independent component analysis (ICA) was calculated to remove artifacts of physiological activity caused by eye movements and muscle movements. The reconstructed independent components were assessed as follows (27):

$$\left|∆P|=|P_{\text{ICA}}-{\text{P}}_{\text{raw}}\right|$$

where P is the change of power spectrum between reconstructed data and raw data. The EEG preprocessing of ICA emerged less spectral distortions. The EEG data were subsequently segmented in a time window of 300 ms prestimulus to 800 ms poststimulus onset. The EEG epochs were extracted from the three conditions respectively, which included self-referential condition, other-referential condition, and physical condition.

### Time-Frequency Power Analysis

In each condition, 70 trials were averaged for the evoked power (28) (time-locked and phase-locked spectral power). To investigate the time-frequency distribution (TFD) of evoked power in the three conditions in the healthy controls and schizophrenia patients, the frequency bands of EEG data were transformed *via* a complex Morlet wavelet with the MATLAB wavelet toolbox (MathWorks). The wavelet family employed was defined as f0/sf = 7, with f0 ranging from 0.1 to 30 Hz in 0.5 Hz steps. The TFDs of power were calculated for each condition in each participant. To characterize the evoked power, the averages for each 200 ms time window from −200 to 600 ms were calculated for each participant in the three conditions. Finally, the differences between the groups were converted into topography graphs by p-value.

### Phase Synchronization Connectivity Analysis

The EEG data from each epoch in the three conditions were filtered into the alpha frequency band (8–13 Hz). The instantaneous phase value was calculated through Hilbert transform in a time series of each epoch. Due to the distortions generated in the processing of the Hilbert transform, the first or last 100 ms (50 sample points) were not presented in the results. The PLI was calculated by the instantaneous phase difference (Δϕ) over the time series in each trial (t_n_) as follows:

$$PLI_{ij}(t)=\frac{1}{N}\left|\sum_{n=1}^{N}sign(∆\phi (t_{n}))\right|$$

Each electrode pair (i and j), time, t (ms), trials (n = 1…N), and phase synchronization strengths (PLI value) were obtained for each frequency band and each participant.

### Analysis of Dynamic Network Topology Properties

The functional connectivity of the brain network was measured through the PLI for each pair of electrodes during the time window as previously described in the analysis. To characterize the dynamic network properties connected with the SRM task, a brain network (59 × 59) was constructed by the PLI of the alpha band for each participant in each condition with graph theoretical network analysis (GRETNA) (29) global threshold methods (0.1 ≤ S ≤ 0.5) during the time window. The brain-network parameters (global efficiency, characteristic path length, and nodal efficiency) were calculated to characterize the dynamic network properties between the healthy controls and schizophrenia patients during the three conditions.

### Statistical Analysis

In this study, statistical analyses were performed by statistical product and service solutions (SPSS) version 20.0 (http://www.spss.com/). An analysis of variance (ANOVA), T-test, correlation analysis and linear regression analysis were conducted for the behavioral and electrophysiological data. Correction for multiple comparisons was based on Bonferroni correction. All statistical comparisons were two-tailed with α = 0.05.

In time-frequency analysis, repeated-measures ANOVAs was conducted for each frequency rhythm (theta band, alpha band, beta band) in each duration of 200 ms over the time window of 200 ms prestimulus to 600 ms poststimulus onset with group (healthy control vs. schizophrenia patients) as the between-subjects factor and task condition (self-referential condition, other-referential condition, physical condition) as the within-subject factors. In addition, independent-samples T-test was conducted for the group in the alpha band during task condition, and the p-value was transformed to topology map.

In phase synchronization connectivity analysis, an ANOVA was conducted for average PLI values in each duration of 200 ms over the time window of 200 ms prestimulus to 600 ms poststimulus onset with group as task condition. An independent-samples T-test was conducted between the active window of 100–300 ms poststimulus onset and baseline of each participant in the time window of 200 ms prestimulus onset in healthy controls and schizophrenia patients respectively and between the group at the active window of 100–300 ms poststimulus onset.

For dynamic network topology property analysis, one-way ANOVA was conducted for the global efficiency parameter and characteristic path length with group as task conditions. The topologies of nodal efficiency were submitted separately by independent-samples T-test for the group during the three conditions.

Additionally, to assess relationships between behavioral variables and electrophysiological data, the correlation analysis between SRM bias score and evoked power, functional connectivity strength of PLI, global efficiency, and characteristic path length of the alpha frequency band was calculated using a two-tailed Pearson correlation test. The linear regression analysis was calculated for SRM bias score (independent variable) with evoked power, functional connectivity strength of PLI, global efficiency, and characteristic path length (dependent variables) in the alpha frequency band. To simplify the results, the effects that did not reach significance were ignored.

## Results

### Behavioral Analysis

The reaction time was conducted with a two-way ANOVA: factors of conditions (self-referential condition, other-referential condition, physical condition) and groups (healthy controls and schizophrenia patients). There was a significant main effect of conditions (F_2,34_ = 131.144, p < 0.001) and groups. With no significant interaction effect (F_2,34_ = 0.18, p = 0.836), the physical condition (mean ± S.D. = 630.5 ± 101.4 ms) was shorter than the other two conditions (self-referential condition = 999.4 ± 159 ms, other-referential condition = 1,032.9 ± 195.9 ms) (p < 0.001).

**Figure 2:**
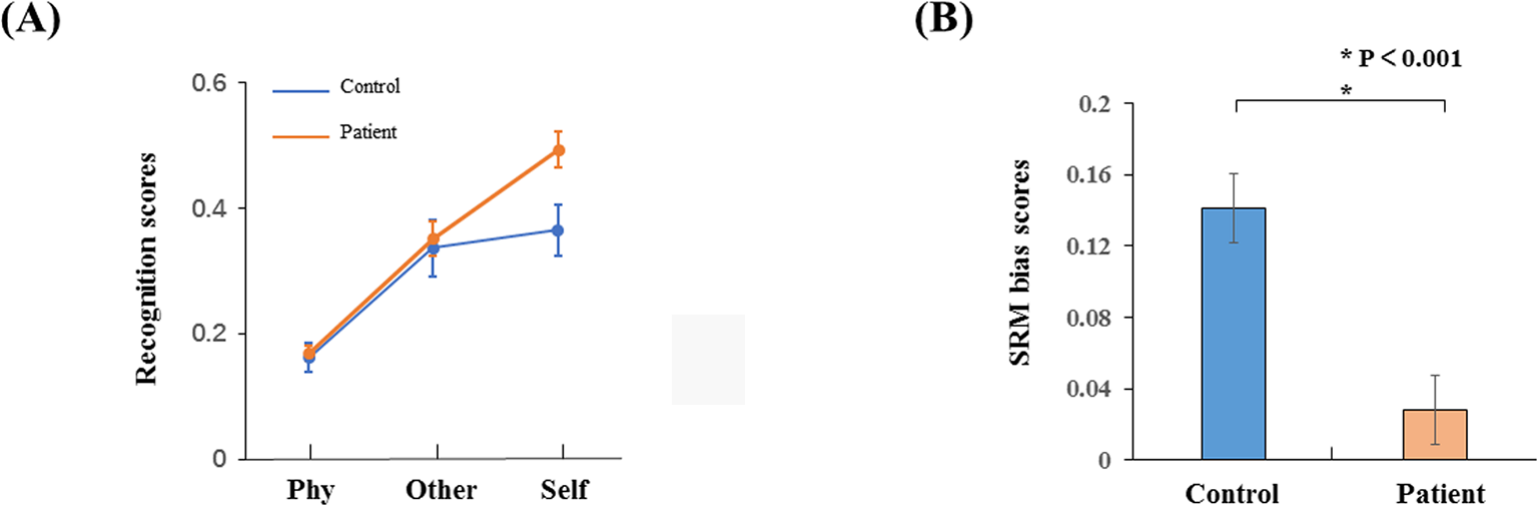
Behavior results. **(A)** Recognition phase with schizophrenia patients and healthy controls in three conditions (self-referential condition, other-referential condition, physical condition). **(B)** SRM bias scores in schizophrenia patients and healthy controls. Error bars are standard error (SE), and black asterisks indicate significant differences (P < 0.001).

The recognition scores were conducted with a two-way ANOVA: factors of conditions and groups. There was a significant interaction effect (F_2,34_ = 9.676, p < 0.001) as shown in **Figure 2A**. The recognition scores for the healthy controls (mean ± S.E. = 0.49 ± 0.03) were higher than the schizophrenia patients in the self-referential condition (0.36 ± 0.04, p = 0.014). There was no significant difference in other-referential condition (p = 0.78) or physical condition (p = 0.767). The recognition scores for the healthy controls in the self-referential condition were higher than in the other-referential condition (0.35 ± 0.03, p < 0.001). In contrast, the recognition scores for the schizophrenia patients were not significantly different between the self-referential condition and other-referential condition (p = 0.459). Therefore, healthy controls reflected a reliable SRM effect. Furthermore, the SRM bias scores for the healthy controls (0.14 ± 0.08) were higher than the schizophrenia patients (0.03 ± 0.08, t_34_ = −4.177, p < 0.001) by independent-samples T-test as shown in **Figure 2B**. 

### Evoked Power of Neural Activity

**Figure 3:**
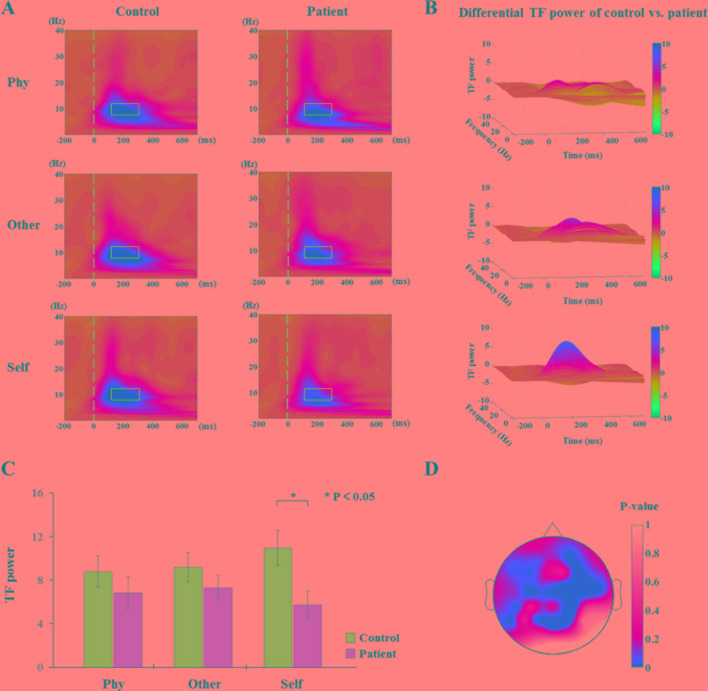
Time-frequency distribution (TFD) of evoked power. **(A)** The average time-frequency power of electrodes at 0.1 to 30 Hz from −200 to 700 ms in the three conditions between healthy controls and schizophrenia patients. The peak value of evoked power region (alpha band: 8–13 Hz, 100–300 ms poststimulus onset) is illustrated in alpha band with blue box between groups (control vs. patient). **(B)** TFD of differential time-frequency power of control vs. patient in three-dimensional plot. **(C)** Histogram with average of evoked power (alpha band: 8–13 Hz, 100–300 ms poststimulus onset) for healthy controls and schizophrenia patients during three conditions. Standard error bars denote SEs, and black asterisks indicate significant differences (P < 0.05). **(D)** Topography of significant differences between healthy controls and schizophrenia patients with average of evoked power (alpha band: 8–13 Hz, 100–300 ms poststimulus onset) in each electrode during self-referential condition by p-value.

The results of the time-frequency distribution of evoked power are shown in **Figure 3A**. The differential time-frequency power was calculated as shown in **Figure 3B**. There was a larger difference in the time window of 100–300 ms between groups in the self-referential condition. There were no main effect results for the group and conditions; the results of the sample effect are illustrated in **Figure 3C**. There was a significant difference between groups in the alpha frequency range at the time window of 100–300 ms poststimulus onset during the self-referential condition (F_1,34_ = 6.498, p = 0.015). However, there were no significant group effects in the task conditions and no significant group effects in the theta or beta bands during the three conditions. In the alpha frequency range, the healthy controls exhibited a higher evoked power than the schizophrenia patients in the self-referential condition. Moreover, the topography of significant differences (**Figure 3D**) was demonstrated over frontal and parietal areas between the groups in the alpha band at the time window of 100–300 ms.

### Associations Between SRM Effect and Evoked Power of Alpha Oscillation

The results indicate that the SRM bias score significantly correlated with the evoked power of the alpha frequency band in the schizophrenia patients during the self-referential condition (r = 0.595, p = 0.009). There was no significant correlation between the SRM bias score and the evoked power of the alpha frequency band in the healthy controls. Moreover, the evoked power of the alpha frequency band in the schizophrenia patients during the self-referential condition was significant to express the SRM bias score (F_1,16_ = 8.752, p = 0.009, R^2 =^ 0.354) by linear regression analysis. There was no significant effect in the regression analysis for the healthy controls.

### Time Courses of the Average PLI

**Figure 4:**
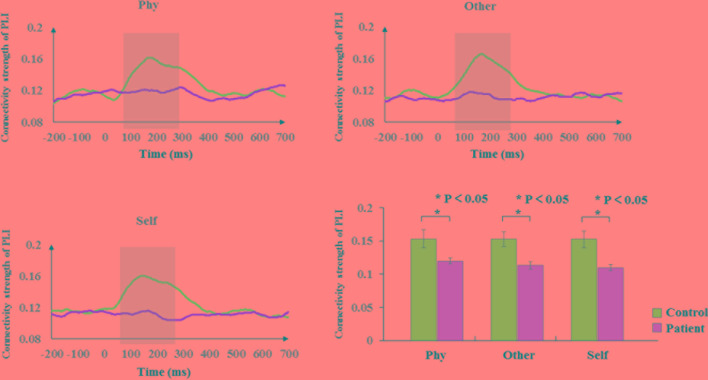
Phase synchronization connectivity of PLI. Time courses of phase synchronization connectivity strength and histogram with average of PLI in the time window of 100–300 ms for healthy controls and schizophrenia patients during three conditions. The gray bar illustrates the time range of significant difference between healthy controls and schizophrenia patients. Standard error bars denote SEs, and black asterisks indicate significant differences (P < 0.05).

According to the time-frequency analysis of evoked power, we focused on the alpha frequency band to characterize the phase synchronization and functional connectivity. The average phase synchronizations of the alpha frequency range were determined for the groups in **Figure 4**. Within-group, the sample effect exhibited a significant difference in the time window of 100–300 ms poststimulus onset during the three conditions (physical: F_1,34_ = 5.207, p = 0.029; other: F_1,34_ = 10.304, p = 0.003; self: F_1,34_ = 10.671, p = 0.002). The healthy controls exhibited a higher functional connectivity strength of the PLI than the schizophrenia patients in the alpha band. No significant group differences were identified in other time windows (p > 0.05).

### Brain-Network Functional Connectivity of PLI

**Figure 5:**
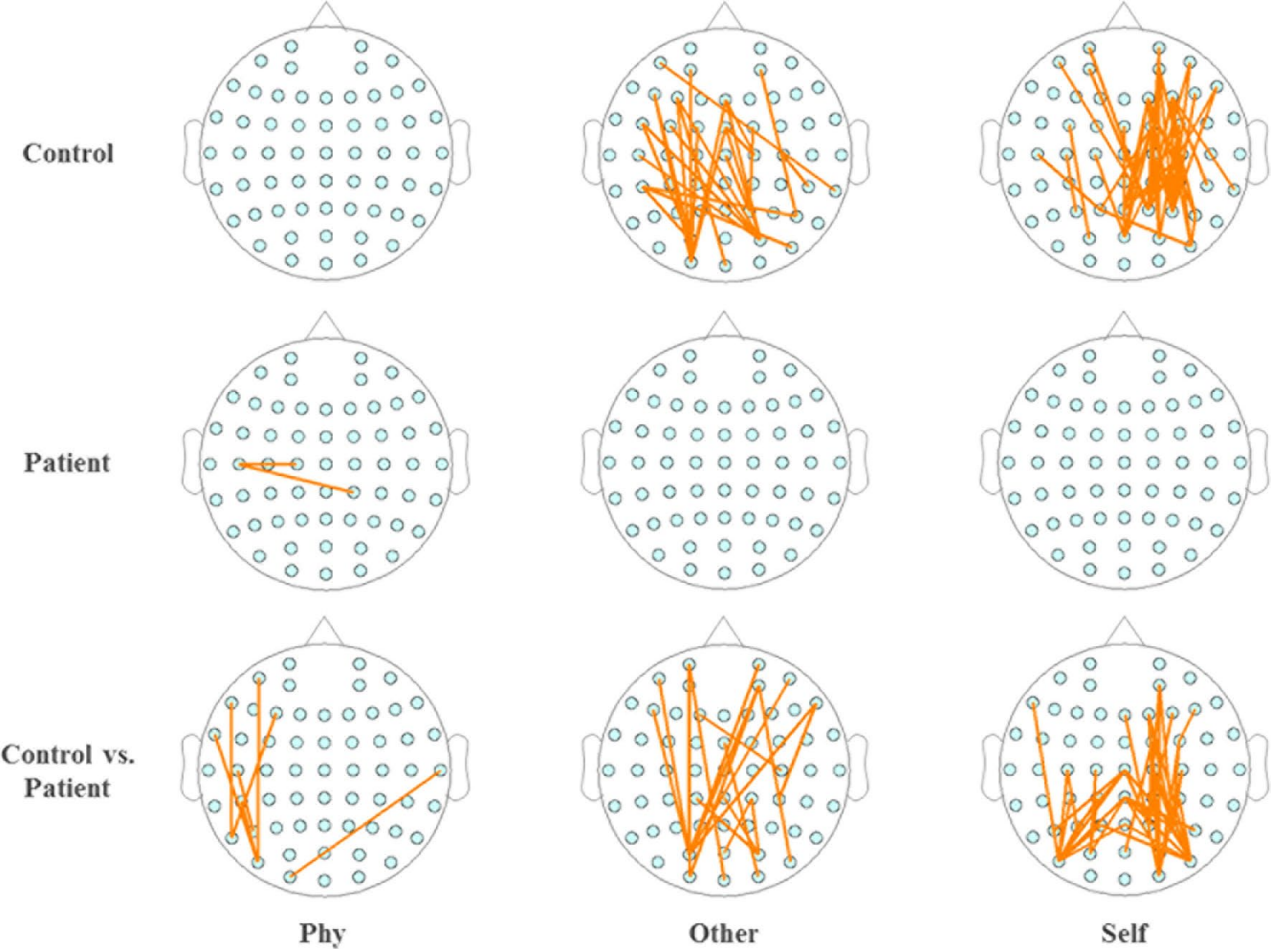
Topography of phase synchronization connectivity. Topography of phase synchronization connectivity for all conditions and time window of 100–300 ms in the alpha band. Top row, healthy controls. Middle row, schizophrenia patients. Bottom row, difference map. In the map of healthy controls and the map of schizophrenia patients, significant differences in the synchronizations between electrodes for the functional connectivity were illustrated by orange lines for the time window of 100–300 ms vs. the average baseline of −200 to 0 ms (p < 0.001), respectively. In the difference maps in the bottom row, significant differences in functional connectivity are illustrated by orange lines for groups (control vs. patient).

Within the healthy controls and schizophrenia patients, the topography of functional connectivity illustrated a significant difference in functional connectivity (p < 0.001) between the active window of 100–300 ms poststimulus onset and the average baseline of each participant in the time window of 200 ms prestimulus onset by independent-samples T-test. Reduced connectivity was demonstrated in the schizophrenia patients compared with the healthy controls during the other-referential condition and self-referential condition. Accordingly, there were significant differences in the functional connectivity between the healthy controls and schizophrenia patients during the other-referential condition and self-referential condition in the difference maps of **Figure 5** by independent-samples T-test at an active window of 100–300 ms poststimulus onset. Specifically, the schizophrenia patients exhibited significantly decreased functional connectivity for the central parietal, temporal, and occipital regions during the self-referential condition.

### Theoretical Analysis of Dynamic Network Topology Parameters

**Figure 6:**
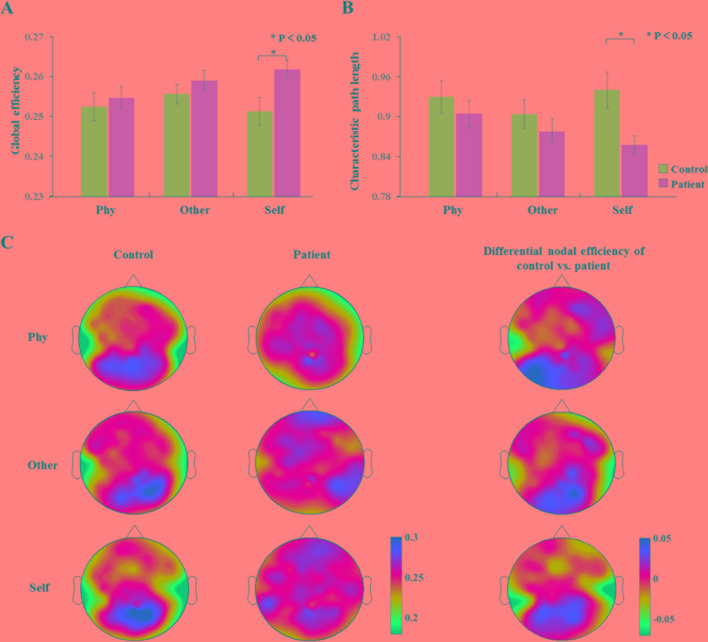
Dynamic network topology properties. **(A**, **B)** Histogram with global efficiency and characteristic path length at the time window of 100–300 ms for healthy controls and schizophrenia patients during three conditions. Standard error bars denote SEs, and black asterisks indicate significant differences (P < 0.05). **(C)** Topography of nodal efficiency for all conditions (top row, front; middle row, other; bottom row, self) with group (first column, healthy controls; second column, schizophrenia patients) at the time window of 100–300 ms in the alpha band. The third column illustrates the difference value with control minus patient. The difference maps indicated increased nodal efficiency for central parietal and temporal regions and decreased nodal efficiency for occipital region in schizophrenia patients compared with healthy controls.

The analysis of the global efficiency (**Figure 6A**) index indicated a significantly increased intensity (F_1,34_ = 6.224, p = 0.018) and significantly depressed (F_1,34_ = 7.353 p = 0.01) characteristic path length (**Figure 6B**) for the schizophrenia patients compared to the healthy controls during the self-referential condition. In the other-referential condition and physical condition, there were no significant main effects for the group. Furthermore, the nodal efficiency was analyzed with topology graphs for the group and the difference value with control minus patient during the three conditions (**Figure 6C**). There was an enhanced nodal efficiency over the occipital area in the healthy controls from the topologies of the differential nodal efficiency. From the topologies of the nodal efficiency, the differences are indicated as follows: increased nodal efficiency of the central parietal region during the three conditions, increased nodal efficiency of the temporal region during other-referential condition and self-referential condition, and decreased nodal efficiency of the occipital region in the schizophrenia patients compared with the healthy controls.

### Associations Between Evoked Power and Dynamic Network Parameters of Alpha Oscillation

The evoked power of the alpha frequency band in the schizophrenia patients during the self-referential condition was significant to express the global efficiency (F_1,16_ = 19.934, p < 0.001, R^2 ^= 0.555) and characteristic path length (F_1,16_ = 14.862, p < 0.001, R^2 ^= 0.482) by linear regression analysis. There was no significant effect in the regression analysis for the healthy controls.

## Discussion

In this study, we investigated the underlying cognitive functions of neural oscillations by TFD, PLI strengths, phase synchronization connectivity, and brain-network properties in healthy controls and schizophrenia patients with three conditions, including the self-referential condition, other-referential condition, and physical condition. These results are significant to understand the underlying neural mechanisms of alpha oscillation in schizophrenia patients compared to healthy controls.

In TFD of three conditions, the results show increased evoked power in the alpha band at 100–300 ms between healthy controls and schizophrenia patients during the three conditions. Interestingly, significantly lower power of the alpha band in schizophrenia patients was found compared to healthy controls over prefrontal and parietal regions during the self-referential condition. The enhanced alpha power by time-frequency analysis is related to a lower performance of attention inward with visual target detection tasks (30, 31). Moreover, the alpha oscillation is important for top–down processing in different cortical regions (32, 33). Significantly lower power of the alpha band in schizophrenia patients was found compared to healthy controls during the self-referential condition. This result is in accordance with a previous study (34), in which schizophrenia patients showed reduced alpha oscillation compared to healthy controls with a focus on a visual attention mechanism. One potential impairment might generate focused visual attention in schizophrenia. Our results provided evidence of an alpha modulation deficit in the cognitive mechanisms of self-referential processing in schizophrenia patients, which indicates that it may be more difficult for schizophrenia patients to encode self-referential information. Moreover, individuals with schizophrenia must consume more attention resources to recognize and select the adjective related to themselves. Furthermore, the SRM bias score significantly correlated with the evoked power of the alpha frequency band during the self-referential condition in schizophrenia patients. The alpha oscillation may be a marker for investigating the cognitive process of self-referential processing in schizophrenia patients.

The current findings indicate that schizophrenia patients exhibited significantly lower PLI strengths in the three conditions. Based on the topological structure of functional connectivity, the indications of functional connectivity are reflected by event-related PLI in schizophrenia patients during self-referential processing. Significantly lower functional connectivity was demonstrated in the parietal region and occipital area. This investigation is the first study to show lower PLI strengths in the alpha band, as well as lower phase synchronization connectivity in networks constructed by PLI, for schizophrenia patients compared with healthy controls in the SRM task. The results of this study were consistent with the reduced functional connectivity of the lower alpha band in schizophrenia patients during the resting state (35, 36). Furthermore, several studies have investigated abnormalities of functional connectivity in the fronto-parietal region in schizophrenia *via* functional and structural neuroimaging (37, 38). Moreover, our results support the hypothesis of dysfunctions of neural activity in the parietal and occipital regions. The preliminary conclusion of deficits in integration related to the symptoms of schizophrenia patients could be reached. Accordingly, the altered alpha phase synchronization connectivity may be employed as a clinical parameter of neural activity during self-referential processing in schizophrenia patients.

To further explore the abnormal brain networks in schizophrenia patients, the global efficiency, characteristic path length, and nodal efficiency were calculated regarding the global and nodal brain-network properties. The global efficiency of network properties as an index of information transmission is calculated by the efficiency value of all nodes, which implies the transmitability of information in the whole brain. The global efficiency might be as a superior measure of integration (39). The characteristic path length is the shortest length for information transmission between a pair of nodes, and it is also an analysis measure that represents the average connectivity and routing efficiency over the whole brain. The higher global efficiency and lower characteristic path length in the brain network suggested an abnormally enhanced functional integration, which is the cognitive basis of high-level cognitive processing (40). This result is consistent with previous studies, in which enhanced functional integration was identified in schizophrenia patients (41, 42). The increased global efficiency and depressed characteristic path length in schizophrenia were associated with whole brain-network resource consumption, which implies stronger parallel information transfer in schizophrenia patients, thus indicating that this processing may consume more cognitive resources than that of healthy controls. In the result of nodal efficiency, schizophrenia patients exhibited a lower nodal efficiency distributed over the occipital region and a higher nodal efficiency distributed over frontal–parietal regions relative to healthy controls. This result is consistent with a study that identified higher functional connectivity in the inferior frontal gyrus and lower connectivity in the visual areas relative to healthy controls (43). Furthermore, the evoked power of the alpha frequency band in the schizophrenia patients during the self-referential condition was significant to express the global efficiency (F_1,16 _= 19.934, p < 0.001, R^2 ^= 0.555) and characteristic path length (F_1,16_ = 14.862, p < 0.001, R^2 ^= 0.482) by linear regression analysis. It may be possible to explain the differences of evoked power with the global efficiency and characteristic path length. Therefore, the alpha oscillation might represent a potential application to evaluate self-referential processing in schizophrenia patients.

The present study has several limitations that should be considered. Prior to this experiment, the schizophrenia patients received drug treatment for at least 1 month; thus, the effects of antipsychotics may have influenced the results of the behavioral data, TFD, PLI strengths, phase synchronization connectivity, and brain-network properties. Furthermore, the small sample may have led to alterations in the statistical analysis in this study. In future studies, the number of participants should be increased to enhance the statistical power.

In conclusion, the present study investigated abnormalities of alpha modulation during the SRM task in schizophrenia patients at the time of 100–300 ms poststimuli, by neurophysiological analyses that consisted of TFD, PLI strengths, phase synchronization connectivity, and brain-network properties. Depressed alpha modulation was identified in schizophrenia patients during the self-referential condition by neurophysiological analysis with the exception of brain-network properties. In the brain-network properties, schizophrenia patients exhibited higher global efficiency, nodal efficiency of frontal–parietal regions, and lower nodal efficiency of occipital region, characteristic path length parameters. Furthermore, significant associations between the SRM effect and the electrophysiological performance (evoked power) of the alpha oscillation were found. The evoked power of the alpha band could effectively represent the SRM bias score. These results provide evidence and support the hypothesis that an abnormal cognitive mechanism of self-referential processing in schizophrenia patients relies on a depressed alpha rhythm.

## Data Availability

The datasets generated for this study are available on request to the corresponding author.

## Ethics Statement

The studies involving human participants were reviewed and approved by Beijing Huilongguan Hospital. The patients/participants provided their written informed consent to participate in this study.

## Author Contributions

SJ: analyzed and interpreted the data, drafting the article, and give approval for the present version. JW: analyzed data and revised the paper. ML: revised the paper. PH: acquired data and analyzed data. YZ: participated in experiments and analyzed behavioral experiment data. ST: carried out scale evaluation. RG: analysed organized results and revised the paper TY: analyzed organized results and revised the paper.

## Funding

This work was supported by the National Key R&D Program of China (grant number 2018YFC0115400), the National Natural Science Foundation of China (grant numbers 81671776, 61727807, 61633018), the Beijing Municipal Science & Technology Commission (grant numbers Z181100003118007, Z191100010618004) and the Beijing Nova Program (grant number Z171100001117057).

## Conflict of Interest Statement

The authors declare that the research was conducted in the absence of any commercial or financial relationships that could be construed as a potential conflict of interest.
